# Circular Dichroism as a Rapid Method for Analyzing the Binding of a Targeting Ligand to the Surface of Albumin Nanoparticles

**DOI:** 10.3390/ph16101423

**Published:** 2023-10-06

**Authors:** Karolina Kulig, Zuzanna Denisiuk, Małgorzata Kłósek, Aleksandra Owczarzy, Wojciech Rogóż, Łukasz Sędek, Małgorzata Maciążek-Jurczyk

**Affiliations:** 1 Department of Physical Pharmacy, Faculty of Pharmaceutical Sciences in Sosnowiec, Medical University of Silesia in Katowice, 40-055 Katowice, Poland; 2 Department of Microbiology and Immunology, Faculty of Medical Sciences in Zabrze, Medical University of Silesia in Katowice, 40-055 Katowice, Poland

**Keywords:** nanoparticles, albumin, circular dichroism

## Abstract

Circular dichroism (CD) is an excellent and rapid method for analysis of chiral molecules, whose mechanism is based on the absorption of left- and right-hand circularly polarized light. Albumin nanoparticles are biocompatible and easy to modify due to their structure. Tumor cell membranes are among the molecules that direct nanoparticles into the tumor microenvironment, but methods to study them except molecular biology are not well validated yet. The aim of this study was to use circular dichroism as the tool to qualitatively assess ligand binding on the surface of nanoparticles. Human serum albumin (HSA) nanoparticles with encapsulated 5-fluorouracil (5-FU) were coated on MCF-7 cell membranes and subjected to CD analysis. This study was completed using sample and separate 5-FU release analysis. The amount of encapsulated drug in nanoparticles affects the binding of cell membranes on the nanoparticle surface. In addition, it can be suspected that the alpha structure of HSA was mainly used for the interaction, which confirms the effectiveness of using CD as a rapid technique for analyzing ligand-nanoparticle interactions. The release of 5-FU from the nanoparticles proceeds in an uncontrolled manner, making this study in need of further modification and investigation.

## 1. Introduction

Nanoparticles used as carriers of therapeutic substances are subject to constant modification in order to develop the most optimal therapy. The main reason for seeking new solutions to improve the properties of nanoparticles is their short circulation half-life. Both nanoparticles made of synthetic and natural polymers are quickly detected by the immune system. The mechanism of their elimination by immune cells involves opsonization, i.e., coating of nanocarriers by various plasma proteins, facilitating their phagocytosis by macrophages or monocytes. As a result of activation of the specific response, antibodies targeting the nanoparticles can be produced. A second therapeutic problem is the relatively low specificity of nanocarriers to target cells. In order to extend nanoparticles’ circulation time in the bloodstream while accumulating in the target regions, a number of strategies are used, including a camouflage method using cell membranes. The following membranes are used to coat the nanoparticles: erythrocytes, platelets, immune cells and cancer cells [1]. Cancer cell membranes can be used to provide nanoparticles with a lack of immunogenicity and better accumulation in cancer cells. The first feature is a consequence of numerous protein structures present on the surface of tumor cells, including the camouflaging CD47 molecule that makes tumor cells difficult to recognize by the immune system. Moreover, the membrane surface is rich in antigens responsible for tumor cell adhesion, such as E-cadherin. Nanoparticles coated with the membrane of tumor cells will therefore exhibit the ability to actively target solid tumors, as well as individual cancer cells circulating in the blood. The properties of such developed nanoparticles make them an attractive tool to be used in targeted therapy and immunotherapy [1].

Albumins are one of the most commonly used proteins to form nanocarriers, as therapeutic substance delivery systems. Human serum albumin (HSA) as well as bovine serum albumin (BSA) are characterized by biocompatibility, biodegradability and low immunogenicity [2]. There are many potential binding sites on the albumin molecule, which creates the possibility to interact with a variety of chemicals, including drugs, cross-linking factors and targeting ligands [2,3].

Circular dichroism (CD) is a sensitive method for rapidly studying the secondary structure of proteins. Due to the differences in absorption of left- and right-hand circularly polarized light, different CD spectra of particular structural elements are obtained [4]. For example, α−helical structures have a negative band at 222 nm and 208 nm, whereas the positive band is about 193 nm [4]. The CD technique also enables the analysis of the secondary structure of recombinant proteins, as well as protein–ligand interactions, among many other applications [4,5].

The aim of this study was to evaluate CD as a rapid method of analyzing the binding of the cell membrane components to the surface of human serum albumin nanoparticles with encapsulated 5-FU. Spectroscopy is a field that allows the evaluation of interactions of drugs with proteins [6] and their delivery systems [7]. Currently for these purposes, UV-vis spectroscopy, FT-IR spectroscopy, zeta potential measurements and electron microscopy are most commonly used [6,8,9,10]. The aim of the present work was to evaluate CD spectroscopy as an alternative technique for studying protein–nanoparticle–ligand interactions, by observing the changes in the secondary structure of the protein. 

## 2. Results

### Circular Dichroism (CD) Measurements

The mean residue ellipticity (Θ_MRW_) and the percentage content of the secondary structure elements are provided in Table 1 and Table 2, respectively.

**Figure 1:**
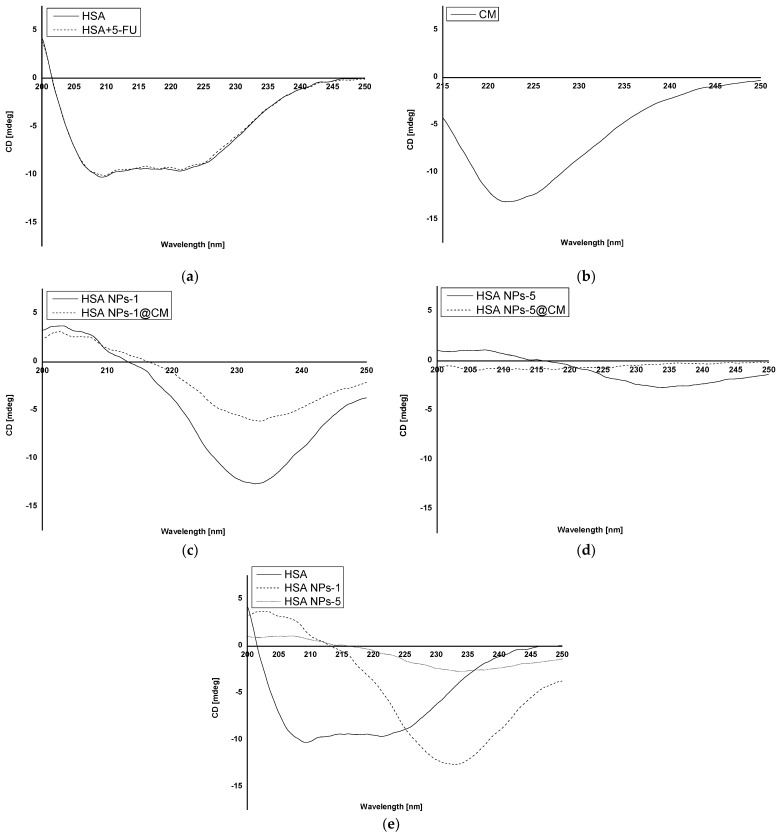
CD spectra of (**a**) native HSA (HSA) and 5-FU + HSA; (**b**) cell membrane; (**c**) HSA NPs-1 and HSA NPs-1@CM; (**d**) HSA NPs-5 and HSA NPs-5@CM; and (**e**) native HSA (HSA), HSA NPs-1 and HSA NPs-5.

Circular dichroism (CD) was performed to study the secondary structure of the obtained nanoparticles, and it shows changes compared to albumin-bound 5-FU (Figure 1a–e).

**Figure 2:**
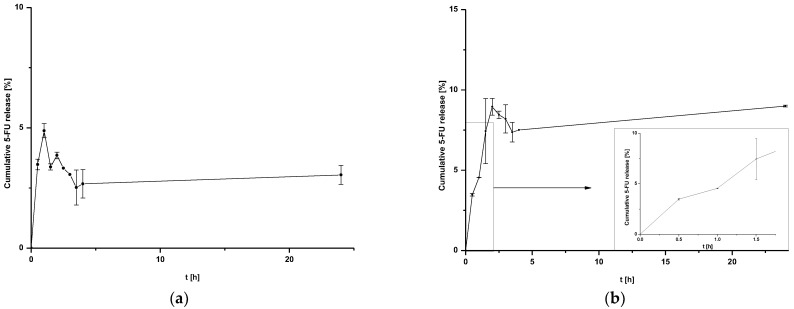
Release of 5-FU from (**a**) HSA NPs-1@CM nanoparticles at 24 h after time T_0_ and (**b**) HSA NPs-5@CM nanoparticles during 24 h after time T_0_ with linear waveform fitting for the first stages of release.

The results of 5-FU release from nanoparticles are shown in Figure 2a,b. Coated nanoparticles were not exposed to additional conditions, such as laser irradiation.

The release of 5-FU from coated nanoparticles proceeds with low efficiency for both HSA NPs-1@CM (Figure 2a) and HSA NPs-5@CM (Figure 2b). In addition, the release profile of the HSA NPs-5@CM sample can be divided into two stages—the first from 0 to 1.5 h, characterized by linearity, and the second, in which the establishment of an equilibrium state with large standard deviations can be observed. This phenomenon is related to an extra barrier to the drug being released.

## 3. Discussion

Coating nanoparticles with membranes is a relatively new trend that aims to improve the active delivery of nanocarriers to specific cell types. Currently, no validated method has been described in the literature to unambiguously verify the presence of cell membranes on the surface of nanoparticles. In addition, no one has proposed a circular dichroism (CD) analysis yet.

CD analysis allows the identification of secondary structures in the protein. It is often used in the analysis of ligand–protein interactions, but its application in the analysis of the structural change of a polymer after biomaterial preparation is still not popular [7,11,12]. In the present study, we analyzed the interaction of the HSA secondary structure in coated and uncoated albumin nanoparticles for two drug concentrations. Based on the CD spectrum of HSA and HSA solution with 5-FU (Figure 1a), it was shown that the drug substance does not affect the stability of the secondary structure of HSA and that 5-FU does not emit any signal. Analyzing the CD spectrum of HSA NPs-1@CM and HSA NPs-1 nanoparticles (Figure 1c), a significant increase in ellipticity in the far UV range (210–250 nm) for the secondary structure was observed. Once they were released, secondary structures were used to bind the cell membrane to the surface of nanoparticles. In addition, when comparing the spectrum described above (Figure 1c) with the CD spectrum of coated and uncoated albumin nanoparticles of 5 mg·mL^−1^ (Figure 1d), it can be seen that the ellipticity value of the secondary structures was lower for the HSA NPs-5, probably due to the need of binding more drug. Moreover, in the case of coated nanoparticles, one can hardly find any secondary structures (a spectrum close to zero), indicating that the rest of the peptide bonds were utilized for membrane adsorption. Based on the observed differences in the CD spectra of HSA, HSA NPs-1 and HSA NPs-5 (Figure 1e), one can conclude that the desolvation method leads to a reduction in the number of secondary structures, and this phenomenon is also influenced by the high drug concentration. A similar relationship was observed in our previous work, where BSA was used as a polymer with different concentrations of chlorambucil [7].

Based on our results, there was a tendency toward a decreased degree of packing the HSA molecule at 209.2 nm after binding to 5-FU in the complex and the encapsulation of more drug in nanoparticles (Table 1). This was evidenced by an increase in the value of Θ_MRW_ for HSA + 5-FU (−1.16·10^6^ deg·cm^2^·dmol^−1^), as compared to that of native HSA (−1.18·10^6^ deg·cm^2^·dmol^−1^). An opposite relationship was observed for Θ_MRW_ values at a wavelength of 221.2 nm. In addition, the value of Θ_MRW_ at 209.2 nm for HSA NPs-1@CM took on a negative sign, unlike other values obtained for nanoparticles, except for HSA and HSA + 5-FU. The data presented in Table 2 indicate that β−sheet and β−return coils were probably used to bind the drug and crosslink using glutaraldehyde, which is also in line with our previous results [6]. Measurements performed for the MCF-7 cell membrane solution were used as a control (Figure 1b). The lack of data on the precise quantitative and qualitative composition of the cell membrane disqualifies it from the calculations and other analyses. Different methods of analyzing the surface and structure of nanoparticles can also be found in the literature. For example, Wen et al. [13] used a transmission electron microscope to study the surface of nanoparticles coated with the erythrocyte membrane.

**Figure 3:**
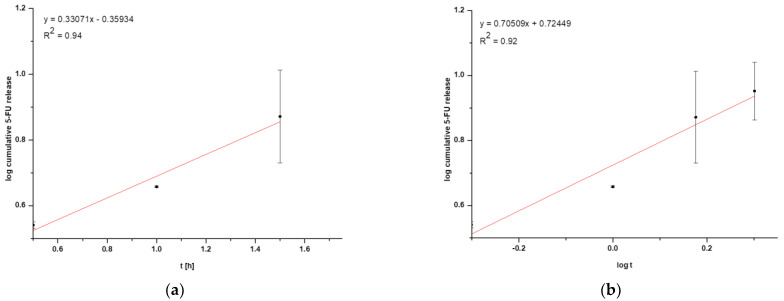
Graphical representation of (**a**) first-order kinetics for HSA NPs-5@CM nanoparticles and (**b**) the Korsmeyer–Peppas model for HSA NPs-5@CM nanoparticles.

In general, coated nanoparticles are characterized by a low release efficiency. This phenomenon may be related to an additional barrier to the drug being released, indicating the need for special conditions, such as heating the target tissues or using laser irradiation [9,13]. It can be seen that the amount of cumulative drug released is at a constant level (within standard deviations) for HSA NPs-1@CM (Figure 2a) and from 1.5 h for HSA NPs-5@CM (Figure 2b). Since the quantitative measurement method allowed a more accurate estimation of the amount of drug released, the points on the graph do not line up in a straight line. The present study was designed to test drug release under basic conditions, without the application of additional stimuli. A similar study was conducted by Cai et al. [14] who synthesized paclitaxel-loaded poly(lactic-co-glycolic) acid (PLGA) nanoparticles coated with the 143B-RAW hybrid membrane and studied the drug release at different pH conditions. Interestingly, the results showed a higher amount of paclitaxel released from coated nanoparticles compared to uncoated ones. Moreover, a higher amount of drug released at pH 5.3 than at pH 7.4 [14]. In another study, atorvastatin was released from ROS-responsive chitosan oligosaccharide NPs conducted in the presence of H_2_O_2_. The concentration of H_2_O_2_ affected the release rate and approximately 20% of the drug was released without peroxide treatment [15]. The release of the drug from the HSA NPs-5@CM in the first 1.5 h was linear in time (Figure 2b), which enabled this part of the release to be presented on graphs using the kinetic equation and Korsmeyer–Peppas model. Based on the calculated values, it was found that the 5-FU release may be characterized by first-order kinetics (Figure 3a); however, it was difficult to definitively confirm this mechanism based on in vitro studies and theoretical assumptions [16]. In contrast, the values obtained for HSA NPs-1@CM after logarithmization did not show the linearity, so no fitting attempt was made. Analysis of the diffusional exponent value based on the Korsmeyer–Peppas model (Figure 3b) developed from the power law suggests a non-Fickian transport mechanism (0.45 < n ≤ 0.85) [16]. Park et al. [17] observed a burst release of dexamethasone from genetically engineered C1498-WT- and engineered C1498-VLA-cell-membrane-coated polymeric nanoparticles in the first hour of the experiment. In addition, the release model studied herein differed from the Peppas–Sahlin model with a regression coefficient of 0.978 [17]. Differences in the obtained results and the literature data may be caused by different cell lines and differences in the method of membrane coating.

## 4. Materials and Methods

### 4.1. Materials

Human serum albumin (HSA), fraction V (purity minimum 96%) and 5-fluorouracil (5-FU) were obtained from Sigma Aldrich (Steinheim, Germany). Ethanol was supplied by P. P. H “STANLAB” Sp. z o. o. (Lublin, Poland). Glutaraldehyde and MgCl_2_ were purchased from Warchem (Warsaw, Poland). Tris(hydroxymethyl)aminomethane (TRIS), HCl and KCl were obtained from POCH (Gliwice, Poland). Protease inhibitor and Dulbecco’s Phosphate Buffered Saline (DPBS) were purchased from Thermo Fisher Scientific (Waltham, MA, USA). MCF-7 cell culture was obtained from the American Type Culture Collection (ATCC, Manassas, VA, USA). All chemicals were of analytical grade and used without further purification.

### 4.2. Preparation of HSA NPs

5-FU solutions were prepared in phosphate buffer (50 mM, pH 7.4) to obtain two solutions of concentrations 1 mg·mL^−1^ and 5 mg·mL^−1^. HSA nanoparticles were prepared using the desolvation method. An amount of 20 mg of HSA was dissolved in 2 mL of 5-FU solutions in order to prepare two separate groups of samples: HSA NPs-1 and HSA NPs-5. Samples were incubated and magnetically stirred at 550 rpm for 15 min with the HSA solutions. After incubation, 96% ethanol was added to reach 8 mL of ethanol per sample. To achieve cross-linked nanoparticles, 4.7 µL of 8% aqueous glutaraldehyde solution was added [6]. The process was performed for 24 h with magnetic stirring. The suspension obtained was purified 2 times via centrifugation in distilled water, 20 °C, 12,000 rpm, and then redispersed via vortex and ultrasonication. Blank nanoparticles (HSA NPs) were prepared according to the procedure presented but without the drug. All steps were performed at room temperature. Samples were frozen with the use of Labconco FreeZone (A.G.A. Analytical, Warsaw, Poland).

### 4.3. In Vitro Culture Conditions and Membrane Isolation

MCF-7 cell lines were cultured in vitro in bottles with a growth area of 75 cm^2^, according to the manufacturer’s recommendations [18], until 80% confluence was achieved. Cell membranes were isolated according to the protocol presented in the literature [17]. To detach the cells from the surface of the bottle, a sterile cell scraper was used, centrifuged (10 min, 125× *g*) and washed twice with DPBS solution. Then, cells were washed with hypotonic solution (20 mM Tris-HCl, pH 7.4; 10 mM MgCl_2_; 10 mM KCl; protease inhibitor) and left in hypotonic buffer for overnight incubation at 4 °C. The cells were centrifuged for 20 min at 20,000× *g*, and the resulting supernatant was again centrifuged at 100,000× *g* for 40 min.

### 4.4. Preparation of Coated HSA NP Cell Membrane

Cell membrane coating was performed according to the literature [19] with modification. Nanoparticle solutions were purified via centrifugation (20 °C, 12,000 rpm) in order to perform the binding process in phosphate buffer (pH 7.4; 0.05 M). Cell membrane solution and nanoparticle suspension (HSA NPs-1, HSA NPs-5) were mixed in a 2:1 ratio and sonicated for 5 min. Coated nanoparticle solutions were purified via centrifugation (20 °C, 12,000 rpm) to remove unbound cell membranes.

### 4.5. Drug Release Study

In vitro 5-FU release from HSA nanoparticles was studied with a sample and separate method in phosphate buffer (pH 7.4; 0.05 M). An amount of 90 mL of each sample was incubated for 24 h at 37 °C under constant gentle stirring (220 rpm) to mimic physiological conditions. The samples were collected at arbitrary time intervals (0.5, 1, 1.5, 2, 2.5, 3, 3.5, 4, 4.5 and 24 h), filtered through filters with a pore diameter of 0.22 µL (Millex-MP, Dublin, Irland) and refilled with the same amount of fresh buffer. 5-FU released from nanoparticles was analyzed with the use of UV-vis spectroscopy using a Jasco V-760 spectrophotometer (Hachioji, Tokyo, Japan). A standard calibration curve was plotted to correlate the absorbance maximum (λ_max_ 267 nm) and the concentration of free 5-FU (R^2^ > 0.99).

To determine the in vitro kinetics and the release mechanisms of 5-FU, the first-order model (Equation (1)) and Korsmeyer–Peppas model (Equation (2)) were used.

(1) $$\frac{\mathrm{dC}}{\mathrm{dt}}=-K_{1}C$$

where C is the concentration of drug and K_1_ is the first-order rate constant expressed in units of time^−1^ [16].

(2) $$\frac{M_{t}}{M_{\infty }}=K_{\mathrm{KP}}\cdot t^{n}$$

where $\frac{M_{t}}{M_{\infty }}$ is the amount of released drug at time t, t is the release time, K_KP_ is the Korsmeyer–Peppas release constant and n is the diffusional exponent indicating the transport mechanism [17].

### 4.6. Circular Dichroism (CD) Measurements

Circular dichroism (CD) spectra of HSA nanoparticles in the presence (HSA NPs-1, HSA NPs-5) and absence of 5-FU (HSA NPs), CM, CM-coated HSA nanoparticles in the presence of 5-FU (HSA NPs–1@CM, HSA NPs–5@CM), native HSA (HSA) and native HSA in the presence of 5-FU (HSA + 5-FU) were recorded using a JASCO J-1500 spectropolarimeter (Hachioji, Tokyo, Japan). The spectrum of free 5-FU was recorded as a control (data not shown). The measurements were conducted at 20 °C in quartz cuvettes with an optical path of 1 mm. The spectra were recorded in the wavelength range of 200 to 250 nm (secondary structure image). The accuracy of the wavelength measurement was ±0.1 nm, the wavelength repeatability was ±0.05 nm and the scanning speed was 20 nm/min.

The percentage (%) content of HSA secondary structure elements based on Yang’s reference model was calculated based on Spectra Manager software.

The mean residue ellipticity [Θ]_MRW_ is represented by Equation (3).

(3) $${\left[\Theta \right]}_{\mathrm{MRW}}=\frac{\mathrm{MRW}\cdot \Theta }{10\cdot l\cdot c}$$

where Θ is the observed ellipticity for a given wavelength (deg), c is the concentration (g/cm^3^), l is the pathlength (cm) and MRW is the mean residue weight (MRW_HSA_ = 114.7 Da).

### 4.7. Data Analysis and Statistics

All samples were performed in at least duplicate. The results of the measurements carried out are expressed as arithmetic mean ± standard deviation (SD). In order to perform calculations and graphical interpretation of the results, Origin Pro software version 8.5 SR1 was used. For CD analysis, data were calculated based on Spectra Manager software version 2.13.00 2002–2015.

## 5. Conclusions

Albumin, due to its complex structure and high content of secondary structures, is an excellent carrier for 5-FU. The results show that CD analysis is an effective method for the initial identification of the presence of a membrane on the surface of coated nanoparticles. Secondary structures are responsible for the binding of 5-FU and cell membranes by albumin. Regardless of the concentration, uncoated albumin nanoparticles have the same release mechanism. 5-FU is released from coated albumin nanoparticles with lower efficiency compared to uncoated nanoparticles. In addition, HSA NPs-1@CM release 5-FU in an uncontrolled pattern.

## Figures and Tables

**Table 1:** The mean residue ellipticity (Θ_MRW_) of HSA secondary structure elements.

	Θ_MRW_ at 209.2 nm (deg·cm^2^·dmol^−1^)	Θ_MRW_ at 221.2 nm (deg·cm^2^·dmol^−1^)
HSA	−1.18·10^6^	−1.11·10^6^
HSA + 5-FU	−1.16·10^6^	−1.09·10^6^
HSA NPs-1	1.82·10^5^	−5.45·10^5^
HSA NPs-5	8.68·10^4^	−9.24·10^4^
HSA NPs-1@CM	1.86·10^5^	−2.03·10^5^
HSA NPs-5@CM	−9.74·10^4^	8.10·10^4^

**Table 2:** The percentage (%) content of HSA secondary structure elements based on Yang’s reference model.

	% α−Helix	% β−Sheet	% Turn	% Random
HSA	35.9	15.7	19.3	29.2
HSA + 5-FU	34.6	16.2	19.8	29.4
HSA NPs-1	96.0	0	0	4.0
HSA NPs-5	100.0	0	0	0
HSA NPs-1@CM	100.0	0	0	0
HSA NPs-5@CM	17.0	15.5	25.5	41.9

## Data Availability

Data sharing is not applicable to this article.
